# Proteome-wide Changes in the *mdx-4cv* Spleen due to Pathophysiological Cross Talk with Dystrophin-Deficient Skeletal Muscle

**DOI:** 10.1016/j.isci.2020.101500

**Published:** 2020-08-26

**Authors:** Paul Dowling, Stephen Gargan, Margit Zweyer, Michael Henry, Paula Meleady, Dieter Swandulla, Kay Ohlendieck

**Affiliations:** 1 Department of Biology, Maynooth University, National University of Ireland, Maynooth, Co. Kildare W23F2H6, Ireland; 2 Kathleen Lonsdale Institute for Human Health Research, Maynooth University, Maynooth, Co. Kildare W23F2H6, Ireland; 3 Department of Neonatology and Paediatric Intensive Care, Children's Hospital, University of Bonn, 53113 Bonn, Germany; 4 National Institute for Cellular Biotechnology, Dublin City University, Dublin 9, Ireland; 5 Institute of Physiology II, University of Bonn, 53115 Bonn, Germany

**Keywords:** Disease, Pathophysiology, Proteomics

## Abstract

Duchenne muscular dystrophy is primarily characterized by progressive muscle wasting due to deficiency in the membrane cytoskeletal protein dystrophin but is also associated with body-wide cellular disturbances in a variety of non-muscle tissues. In this study, we have focused on the comparative proteomic analysis of the spleen and established considerable changes in this crucial secondary lymphoid organ from the genetic *mdx-4cv* mouse model of dystrophinopathy. An apparent short isoform of dystrophin and associated glycoproteins were identified in spleen by mass spectrometry but appear not be affected in muscular dystrophy. In contrast, the *mdx-4cv* spleen showed significant proteome-wide changes in other protein species that are involved in metabolism, signaling, and cellular architecture. Since the spleen plays a key role in the immune response, these proteomic alterations may reflect pathophysiological cross talk between the lymphoid system and dystrophic muscles, which are affected by both fiber degeneration and inflammation.

## Highlights

• Biochemical and proteomic analyses have identified dystrophin isoform Dp71 in spleen
• Normal Dp71 levels were found in the spleen of the *mdx-4cv* model of dystrophinopathy
• The enzymes TGM2 and MMP-9 are drastically increased in *mdx-4cv* spleen
• Apolipoproteins ApoE and ApoB are greatly reduced in *mdx-4cv* spleen

## Graphical Abstract

**Figure:**
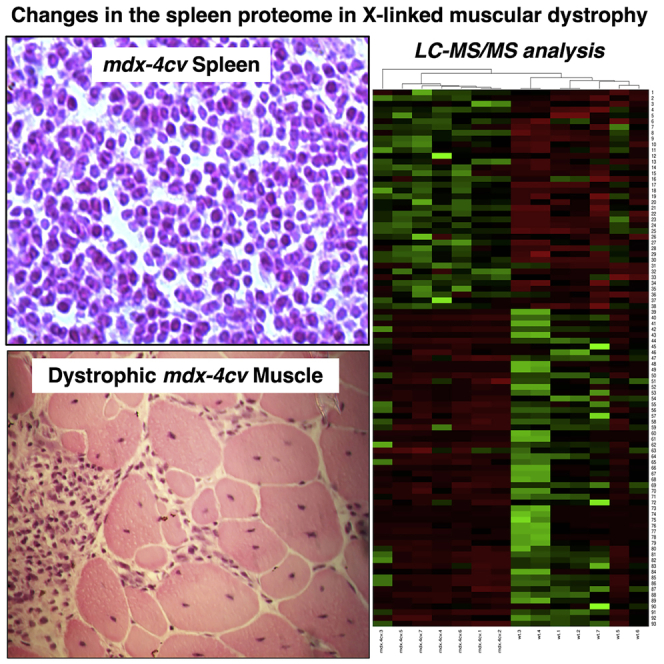

## Introduction

The neuromuscular disorder Duchenne muscular dystrophy is due to primary abnormalities in the *DMD* gene (Guiraud et al., 2015), which encodes several different isoforms of the protein dystrophin (Muntoni et al., 2003). Large-scale and antibody-based surveys of the tissue-specific presence of individual proteins provide excellent information on the distribution of protein markers throughout the body (Thul and Lindskog, 2018). However, often the reliance on a single, albeit highly specific, antibody for antigen detection does not take into account the existence of multiple protein isoforms with greatly differing cellular expression patterns. This is especially apparent in the case of the extremely large 79 exon-spanning *DMD* gene, which contains seven promoters that drive the tissue-specific expression of various dystrophin isoforms of differing molecular mass (Muntoni et al., 2003). This includes three full-length isoforms of apparent 427 kDa, i.e., Dp427-M in contractile tissues, Dp427-B in the brain, and Dp427-P in Purkinje cells. Shorter dystrophin isoforms of approximately 116–260 kDa are represented by Dp116-S in Schwann cells, Dp140-B/K in brain and kidney, and Dp260-R in the retina. In addition, an ubiquitous proteoform named Dp71-G exists in the brain and various other tissues (Naidoo and Anthony, 2020).

Dystrophinopathies belong to a group of inherited degenerative diseases of muscles (Mercuri et al., 2019; Thompson et al., 2020) that are characterized by progressive changes in the skeletal musculature, including degeneration of contractile fibers, sterile inflammation, fatty tissue infiltration, and reactive myofibrosis (Allen et al., 2016; Dowling et al., 2020a; Shin et al., 2013; Smith and Barton, 2018; Tidball et al., 2018). The recent mass spectrometry-based proteomic profiling of the *vastus lateralis* muscle from patients with Duchenne muscular dystrophy has confirmed severe extracellular and cytoskeletal dysregulation in dystrophinopathy (Capitanio et al., 2020). Additional complications manifest themselves as metabolic abnormalities, hormonal disturbances, scoliosis, neuronal deficiencies, and late-onset cardio-respiratory impairments (Birnkrant et al., 2018; Meyers and Townsend, 2019; Saure et al., 2018). Thus, although X-linked muscular dystrophy is primarily a monogenetic disease of contractile tissues, the complexity of its secondary pathophysiology makes it a neuromuscular disorder with a high degree of vulnerability of other tissue and organ systems.

Animal models of Duchenne muscular dystrophy have been instrumental for studying the molecular and cellular details of progressive degeneration in dystrophin-deficient muscle tissues, as well as testing of novel experimental treatment strategies to counteract the dystrophic phenotype (McGreevy et al., 2015; Rodrigues et al., 2016; Wilson et al., 2017). One of the most frequently used models of dystrophinopathy is the *mdx-23* mouse, which represents a naturally occurring mutant with a premature stop codon-inducing mutation in exon 23 of the *DMD* gene (Sicinski et al., 1989). Hence, in analogy to progressive forms of human dystrophinopathy, this animal model almost completely lacks the full-length dystrophin isoform Dp427-M (Partridge, 2013). An alternative *mdx*-type mouse model (*mdx-4cv*) was generated by chemical mutagenesis with N-ethyl-N-nitrosourea (Chapman et al., 1989) and resulted in a C to T transition at base 7,916 in exon 53, generating an ochre codon (Banks et al., 2010; Shin et al., 2011; Tichy and Mourkioti, 2017). Importantly, the *mdx-4cv* model is typified by an approximately 10-fold lower rate of dystrophin-positive revertant fibers as compared with the conventional *mdx-23* mouse (Danko et al., 1992; Im et al., 1996; Judge et al., 2006). This feature of the genetic mouse model reflects more accurately the cellular characteristics of affected skeletal muscles from patients with Duchenne muscular dystrophy. Consequently the *mdx-4cv* musculature represents an attractive tissue source for the systematic evaluation of new therapeutic approaches, such as exon skipping following intramuscular injections of antisense oligomers (Mitrpant et al., 2009), viral vector injection into neonatal muscles for the stable restoration of dystrophin expression (Kimura et al., 2010), or CRISPR-Cas9-based dystrophin gene editing (Bengtsson et al., 2017).

Previous mass spectrometric investigations focusing on the *mdx-4cv* mouse have established differential changes in the tissue proteome from various skeletal muscles, the heart, the brain, the liver, and the kidney (Murphy et al., 2015, 2016; 2018a, 2019a; 2019b; Dowling et al., 2020b) and have identified elevated levels of biofluid-associated markers in saliva, serum, and urine (Murphy et al., 2017, 2018b; Gargan et al., 2020). In this study, we have extended the proteomic characterization of body-wide alterations in the *mdx-4cv* mouse model of dystrophinopathy to the spleen, a secondary lymphoid organ that consists of two main types of tissue, the blood-filled red pulp and the lymphatic white pulp (Mebius and Kraal, 2005). The main functions of the spleen include the removal of abnormal erythrocytes, antigen detection, and antibody production (Lewis et al., 2019). In dystrophic organisms with an almost complete loss of the full-length Dp427-M isoform of dystrophin, abnormalities in the spleen were previously reported to include morphological adaptations in relation to lymph nodes in the white pulp region of the *mdx* mouse spleen (Santos et al., 2013), as well as altered levels of splenic inflammatory monocytes and increased migration of immune cells from the splenic reservoir to injured dystrophic fibers (Farini et al., 2016; Giordano et al., 2015; Mojumdar et al., 2014, 2016; Ouisse et al., 2019; Rizzo et al., 2020).

The proteomic survey presented here has initially identified the experimentally assessable protein constituents of the mouse spleen with the help of an Orbitrap Fusion Tribrid mass spectrometer. The established splenic protein catalog was screened for the presence of markers of spleen function and the potential presence of dystrophin, which had previously been suggested by the RT-PCR analysis of dystrophin in non-muscle tissues (Tokarz et al., 1998), as well as the more widely distributed class of dystrophin-associated proteins. Comparative mass spectrometric analyses of wild-type versus *mdx-4cv* tissue extracts were then used to determine potential proteome-wide changes due to deficiency in dystrophin isoform Dp427-M. The proteomic findings suggest that a variety of metabolic and cellular processes are affected in the *mdx-4cv* spleen, which confirms the usefulness of this genetic mouse model for studying the complex pathology of Duchenne muscular dystrophy. A pathophysiological connection appears to exist between skeletal muscle wasting, which is characterized by progressive fiber degeneration and inflammation (Allen et al., 2016; Tidball et al., 2018), and alterations in the lymphoid system due to primary abnormalities in the dystrophin gene (Rizzo et al., 2020).

## Results

In order to evaluate the complex pathogenesis of X-linked muscular dystrophy, this study has focused on the proteomic characterization of potential secondary effects in a non-muscle organ, the spleen, in the established *mdx-4cv* mouse model of dystrophinopathy. Prior to the comparative mass spectrometric analysis of this secondary lymphoid organ, the spleen proteome was evaluated for the presence of dystrophin and spleen marker proteins.

### Mass Spectrometric Identification of the Splenic Isoform of Dystrophin

**Figure 1:**
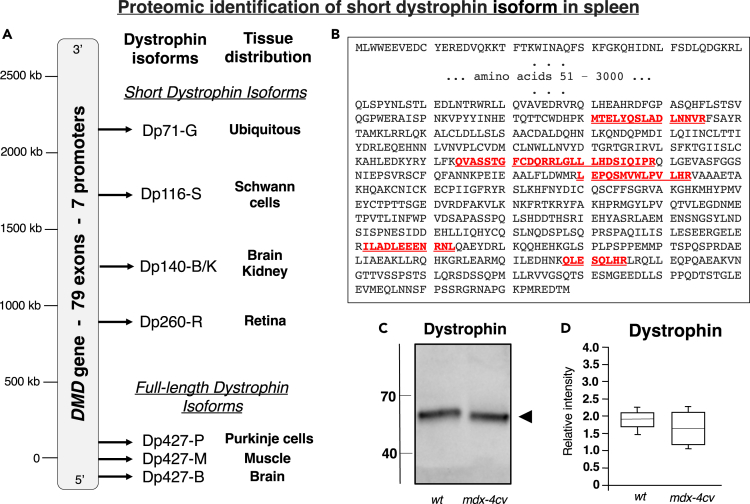
Mass Spectrometric Identification of a Short Isoform of Dystrophin in Mouse Spleen (A) Diagrammatic presentation of the *DMD* gene and its 7 promoters. (B) Overview of unique peptides determined by the mass spectrometric analysis of 12-month-old mouse spleen and their position within the carboxy terminal region of the dystrophin protein sequence (C and D) Comparative immunoblot analysis of the apparent spleen isoform Dp71 of dystrophin in wild-type versus *mdx-4cv* preparations. Lanes 1 and 2 contain wild-type and *mdx-4cv* specimens, respectively (C). In (D) the box plots of the immunoblot analysis are shown (Mann-Whitney U test; n = 5; none significant). The value of molecular mass standards (x10^−3^ kDa) is marked on the left side of the gel. A representative protein gel of spleen extracts used to produce nitrocellulose replicas is shown in Figure 7A.

**Figure 2:**
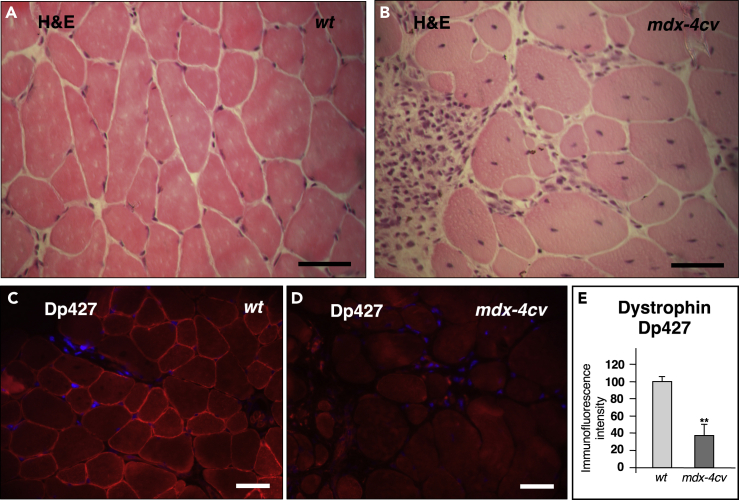
Histological and Immunofluorescence Microscopical Characterization of Skeletal Muscle from the *mdx-4cv* Mouse Model of Duchenne Muscular Dystrophy (A–D) Shown are transverse cryosections of wild-type (*wt*) (A and C) and *mdx-4cv* (B and D) *gastrocnemius* muscle stained with hematoxylin and eosin (H&E) (A and B) and labeled with antibodies to the full-length Dp427 isoform of dystrophin (C and D). In (E) the analysis of immunofluorescence intensities is shown (unpaired Student's t test; mean values ± SEM; n = 4; ∗∗p < 0.01). Dystrophic muscle fibers show abnormal fiber diameters, central nucleation, cellular degeneration, and inflammation, as well as the almost complete loss of dystrophin. Scale bar, 50 μm.

To overcome the restricted specificity of protein isoform coverage by antibody screening using standard immunochemical methodology (Thul and Lindskog, 2018), we have employed here a more sensitive proteomic screening approach with an Orbitrap Fusion Tribrid mass spectrometer for determining the potential presence of low levels of dystrophin in the spleen. As listed in Table 1, dystrophin and various dystrophin-associated proteins were clearly identified by this method, including an apparent short isoform of dystrophin, dystroglycan, beta-sarcoglycan, delta-sarcoglycan, epsilon-sarcoglycan, alpha-dystrobrevin, alpha-1-syntrophin, and beta-1-syntrophin. Dystrophin was recognized by six unique peptides, and these sections of the dystrophin sequence clearly aligned with the domain of the dystrophin protein that is close to the carboxy terminus, as shown in Figures 1A and 1B. Immunoblotting indicated that the expression of this short spleen-associated isoform of dystrophin is not affected in dystrophinopathy (Figures 1C and 1D), but the full-length Dp427-M isoform of dystrophin was clearly shown to be absent from *mdx-4cv gastrocnemius* muscle, as illustrated in Figures 2C–2E. The histological staining of *mdx-4cv* muscle depicts the typical hallmarks of X-linked muscular dystrophy, including variations in fiber diameter, a high degree of central nucleation, fibrosis, and inflammation (Figures 2A and 2B).

**Table 1:** Proteomic Identification of Dystrophin and Dystrophin-Associated Proteins in Mouse Spleen

Accession	Protein	Gene	Coverage (%)	Peptides	Unique Peptides	Molecular Mass (kDa)
P11531	Dystrophin Dp71	Dmd	2	6	6	71
Q62165	Dystroglycan	Dag1	13	10	10	96.8
P82349	Beta-sarcoglycan	Sgcb	5	1	1	34.9
P82347	Delta-sarcoglycan	Sgcd	13	3	3	32.1
O70258	Epsilon-sarcoglycan	Sgce	2	1	1	49.7
Q9D2N4	Alpha-dystrobrevin	Dtna	6	2	2	84
Q61234	Alpha-1-syntrophin	Snta1	6	2	1	53.6
Q99L88	Beta-1-syntrophin	Sntb1	6	3	1	58

### Mass Spectrometric Profiling of the Mouse Spleen Proteome

Prior to carrying out a comprehensive proteome-wide comparison between wild-type and *mdx-4cv* spleen, it was essential to first establish how well the chosen proteomic approach covered the accessible splenic protein constituents using total tissue extracts. The biochemical cataloging of 12-month-old mouse spleen with the help of an Orbitrap Fusion Tribrid mass spectrometer resulted in the identification of 5,688 splenic protein species. This table was deposited as a supplementary multi-consensus file to the Open Science Framework repository under the following link: https://osf.io/f85ve/. In addition, individual files include data from the analysis of 14 separate mass spectrometric sample runs (representing 7 biological repeats of wild-type spleen preparations and 7 biological repeats of *mdx-4cv* spleen preparations, which were also deposited to the OSF entry “f85ve”) and consists of high-confidence peptides that were filtered based on Xcorr values. Importantly, a wide range of spleen-associated proteins were confirmed to be present in the examined tissue extracts.

**Figure 3:**
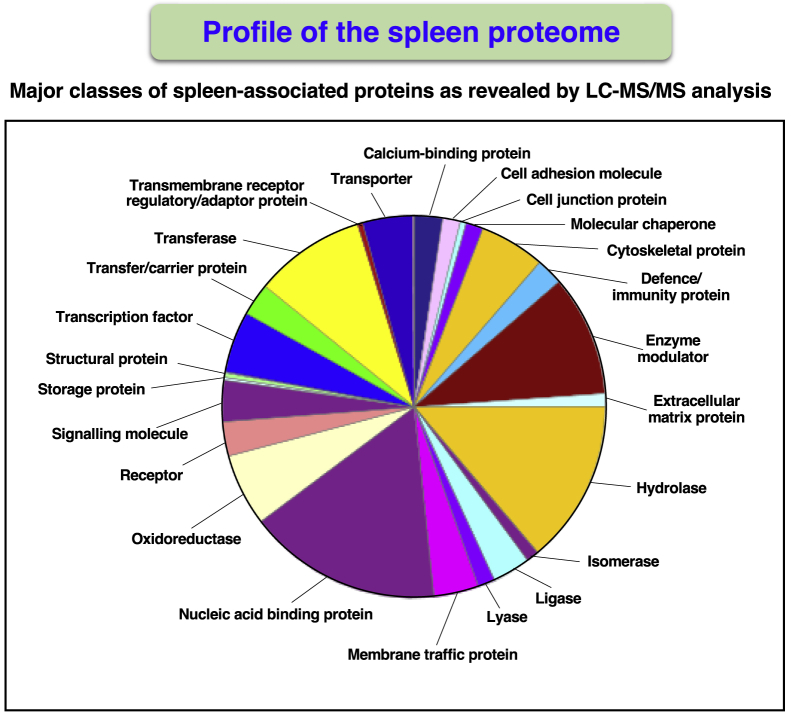
Proteomic Profiling of the Mouse Spleen Shown is the result of the bioinformatic PANTHER analysis of the distribution of protein classes within the accessible proteome from 12-month-old mouse spleen, as determined by LC-MS/MS analysis.

Figure 3 shows a pie chart that summarizes the results from the bioinformatic PANTHER analysis of protein families that were mass spectrometrically identified in mouse spleen preparations. Protein classes included a variety of enzyme families, such as hydrolases, oxidoreductases, and transferases, as well as enzyme modulators, nucleic acid-binding proteins, transcription factors, transporters, and cytoskeletal components. The identification of proteins by the highest percent of sequence coverage agreed with the involvement of the spleen with the removal of abnormal erythrocytes and the immune system. Spleen-associated proteins with a protein sequence coverage above 90% included hemoglobin subunit beta-1 (P02088), hemoglobin subunit beta-2 (P02089), and the immunoglobulin Ig kappa chain (P01654). Splenic proteins that were identified by more than 80 peptides included the crucial cytoskeletal proteins filamin, plectin, talin, myosin-9, myosin-11, dynein heavy chain, erythrocytic alpha/beta spectrin, and non-erythrocytic alpha/beta spectrin, as well as prolow-density lipoprotein receptor-related protein 1 and basement membrane-specific heparan sulfate proteoglycan core protein.

Of note, the proteomic survey of mouse spleen included the identification of major types of splenic receptors, enzymes, and lymphoid proteins, such as cell surface antigens. The spleen marker named CD5 antigen-like protein, which was previously shown to be one of the most highly expressed splenic proteins (Uhlén et al., 2015), was clearly identified as CD5L protein of 38.8 kDa (Q9QWK4; 47% coverage; 14 unique peptides). Other major spleen components that were covered by the mass spectrometric survey included the spleen-associated tyrosine kinase SYK of 71.3 kDa (P48025; 56% coverage; 25 unique peptides), the tyrosine-protein phosphatase non-receptor type substrate 1 SIRPA of 56.4 kDa (P97797; 20% coverage; 7 unique peptides), the abundant spleen marker protein FH1/FH2 domain-containing protein FHOD1 of 129.5 kDa (Q6P9Q4; 19% coverage; 17 unique peptides), the scavenger receptor stabilin STAB2 of 277.3 kDa (Q8R4U0; 20% coverage; 32 unique peptides), CD81 antigen of 25.8 kDa (P35762; 31% coverage; 4 unique peptides), CD82 antigen of 29.6 kDa (P40237; 20% coverage; 5 unique peptides), B-cell receptor CD22 of 96.5 kDa (P35329; 31% coverage; 17 unique peptides), B-lymphocyte antigen CD20 of 31.9 kDa (P19437; 29% coverage; 8 unique peptides), CD209 antigen-like protein B of 37.1 kDa (Q8CJ91; 38% coverage; 10 unique peptides), and CD2-associated protein of 70.4 kDa (Q9JLQ0; 22% coverage; 11 unique peptides).

The screening of the experimentally accessible mouse spleen proteome revealed the coverage of major protein constituents belonging to the family of “classical plasma proteins” (Anderson and Anderson, 2002), as listed in Table 2, including serum albumin; complement factors H, C3, C4-B, C5, C8, and C9; inter alpha-trypsin inhibitor heavy chains 4, H1, H2, and H3; murinoglobulin; haptoglobin; serotransferrin; ferritin light and heavy chains; ceruloplasmin; plasminogen; the alpha, beta, and gamma chains of fibrinogen; plasma kallikrein and von Willebrand factor, as well as the alpha-2-HS, alpha-1B, and beta-2 glycoproteins (Geyer et al., 2017; Moulder et al., 2018; Schwenk et al., 2017). A large number of the crucial class of serum lipid-binding proteins was also covered by the proteomic analysis of the spleen. This included the apolipoproteins of classes A to M, such as apolipoproteins A-I, A-II, A-IV, A-V, B-100, C-I, C-III, C-IV, D, E, F, and M (Table 3). Serum proteins with relevance to muscle diagnostics that are generally defined as “tissue leak markers” (Anderson and Anderson, 2002) were also identified, including myoglobin of 17.1 kDa (P04247; 60% coverage; 10 unique peptides), the muscle-specific Tnnc2 isoform of troponin TnC of 18.1 kDa (P20801; 15% coverage; 1 unique peptide), and M-type creatine kinase of 43 kDa (P07310; 49% coverage; 15 unique peptides).

**Table 2:** Proteomic Identification of Serum Protein Markers in Mouse Spleen

Accession	Protein	Gene	Coverage (%)	Unique Peptides	Molecular Mass (kDa)
P07724	Serum albumin	Alb	73	47	68.6
P06909	Complement factor H	Cfh	56	46	139
P01027	Complement C3	C3	50	67	186.4
P01029	Complement C4-B	C4b	22	28	192.8
P06684	Complement C5	C5	22	27	188.8
Q8K182	Complement C8 alpha	C8a	39	16	66j
P06683	Complement C9	C9	37	19	62
A6X935	Inter alpha-trypsin inhibitor, heavy chain 4	Itih4	34	23	104.6
Q61702	Inter-alpha-trypsin inhibitor, heavy chain H1	Itih1	26	16	101
Q61703	Inter-alpha-trypsin inhibitor, heavy chain H2	Itih2	24	16	105.9
Q61704	Inter-alpha-trypsin inhibitor, heavy chain H3	Itih3	13	8	99.3
P28665	Murinoglobulin-1	Mug1	38	43	165.2
Q61646	Haptoglobin	Hp	35	11	38.7
Q921I1	Serotransferrin	Tf	61	48	76.7
P09528	Ferritin heavy chain	Fth1	70	14	21.1
P29391	Ferritin light chain 1	Ftl1	66	12	20.8
Q61147	Ceruloplasmin	Cp	34	28	121.1
P20918	Plasminogen	Plg	62	33	90.7
E9PV24	Fibrinogen alpha chain	Fga	39	19	87.4
Q8K0E8	Fibrinogen beta chain	Fgb	69	33	54.7
Q8VCM7	Fibrinogen gamma chain	Fgg	64	23	49.4
P26262	Plasma kallikrein	Klkb1	12	6	71.3
Q8CIZ8	von Willebrand factor	Vwf	27	51	309.1
P29699	Alpha-2-HS-glycoprotein	Ahsg	46	10	37.3
Q19LI2	Alpha-1B-glycoprotein	A1bg	27	11	56.5
Q01339	Beta-2-glycoprotein 1	Apoh	46	16	38.6

**Table 3:** Proteomic Identification of Apolipoproteins in Mouse Spleen

Accession	Protein	Gene	Coverage (%)	Unique Peptides	Molecular Mass (kDa)
Q00623	Apolipoprotein A-I	Apoa1	53	19	30.6
P09813	Apolipoprotein A-II	Apoa2	49	6	11.3
P06728	Apolipoprotein A-IV	Apoa4	68	22	45
Q8C7G5	Apolipoprotein A-V	Apoa5	27	8	41.2
E9Q414	Apolipoprotein B-100	Apob	19	69	509.1
P34928	Apolipoprotein C-I	Apoc1	20	2	9.7
P33622	Apolipoprotein C-III	Apoc3	27	2	11
Q61268	Apolipoprotein C-IV	Apoc4	9	1	14.3
P51910	Apolipoprotein D	Apod	6	1	21.5
P08226	Apolipoprotein E	Apoe	63	20	35.8
Q91V80	Apolipoprotein F	Apof	6	1	34.3
Q9Z1R3	Apolipoprotein M	Apom	12	2	21.3

### Proteomic Profiling of the *mdx-4cv* Spleen

**Figure 4:**
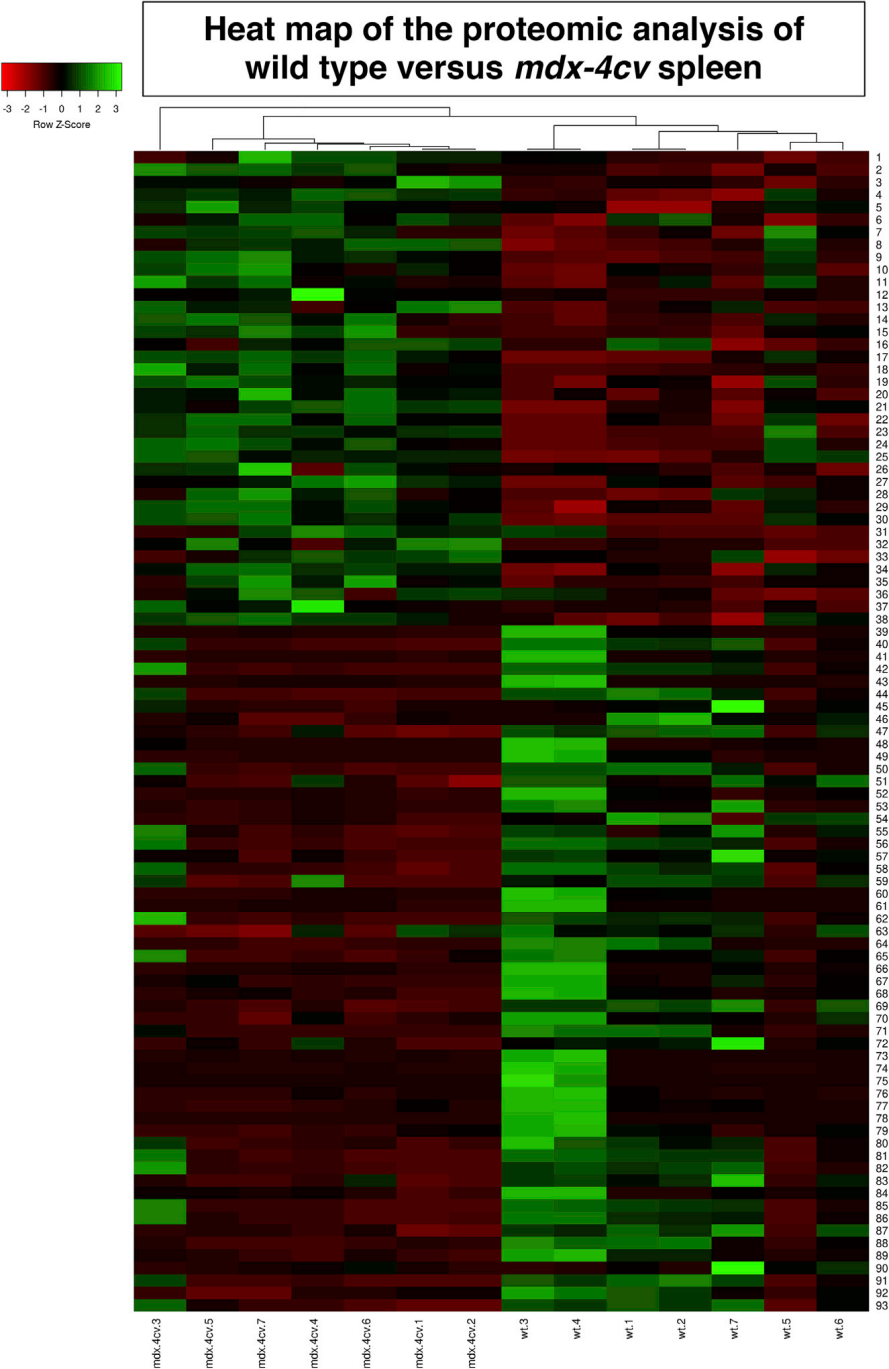
Heatmap of the Comparative Proteomic Analysis of Wild-Type versus *mdx-4cv* Spleen Shown are the findings from hierarchical clustering of the mean protein expression values of statistically significant differentially abundant spleen proteins.

The comparative mass spectrometric survey of wild-type versus *mdx-4cv* spleen extracts identified 10.93% change in protein constituents. Of these components, 93 protein species were identified by a coverage of their amino acid sequence by at least 2 peptides and a minimum fold change of 1.5. A reduced expression was found to occur in 55 proteins, and 38 proteins were shown to be increased in their abundance, as listed in Table 4 and Table 5, respectively. The tables with the findings from the comparative proteomic analysis provide information on accession number, protein name, gene symbol, number of unique peptides, confidence score, adjusted p value, and fold change. In addition to changed proteins that were identified by at least 2 unique peptides, another 159 decreased proteins and 145 increased proteins were identified by only 1 unique peptide (not shown). Owing to their low sequence coverage, these proteomic hits were not included in the subsequent bioinformatic analysis of the *mdx-4cv* spleen. The most decreased and the most increased spleen-associated proteins were identified as apolipoprotein B-100 (E9Q414) and protein-glutamine gamma-glutamyl-transferase TGM2 (P21981), respectively, in *mdx-4cv* preparations. The heatmap of the comparative mass spectrometric survey is provided in Figure 4 and summarizes the differential expression pattern of changed proteins in wild-type versus the *mdx-4cv* spleen.

**Table 4:** Decreased Proteins in the *mdx-4cv* Spleen as Determined by LC-MS/MS Analysis

Accession	Protein	Gene	Unique Peptides	Confidence Score	Adjusted p value	Max Fold Change
E9Q414	Apolipoprotein B-100	Apob	2	5.4283	0.0176	296.2
A6X935	Inter alpha-trypsin inhibitor, heavy chain 4	Itih4	2	4.6726	0.0172	163.3
Q91ZX7	Prolow-density lipoprotein receptor-related protein 1	Lrp1	2	4.9936	0.0453	48.3
P08226	Apolipoprotein E	Apoe	2	7.0306	0.0413	41.2
Q91WT9	Cystathionine beta-synthase	Cbs	4	9.0211	0.0168	21.1
P03987	Ig gamma-3 chain C region	–	5	13.0469	0.0168	20.3
Q6P8U6	Pancreatic triacylglycerol lipase	Pnlip	2	4.9116	0.0285	15.3
P28665	Murinoglobulin-1	Mug1	5	18.5175	0.0216	8.7
P20918	Plasminogen	Plg	5	14.0486	0.0264	8.2
P01027	Complement C3	C3	2	6.9290	0.0265	5.8
Q8K0C5	Zymogen granule membrane protein 16	Zg16	2	4.7425	0.0205	5.3
Q91X79	Chymotrypsin-like elastase family member 1	Cela1	2	6.6288	0.0255	5
Q64285	Bile salt-activated lipase	Cel	2	4.0527	0.0213	4.4
P32261	Antithrombin-III	Serpinc1	4	13.3814	0.0174	4.2
Q9D8U3	Endoplasmic reticulum resident protein 27	Erp27	2	3.8992	0.0173	4.2
Q504N0	Carboxypeptidase A2	Cpa2	4	11.1796	0.0181	4
Q9JK88	Serpin I2	Serpini2	6	15.7950	0.0174	3.7
P07724	Serum albumin	Alb	3	9.5456	0.0140	3.7
Q5BKQ4	Inactive pancreatic lipase-related protein 1	Pnliprp1	5	12.4300	0.0237	3.6
P00688	Pancreatic alpha-amylase	Amy2	5	15.8377	0.0175	3.5
A6H584	Collagen alpha-5(VI) chain	Col6a5	4	12.5493	0.0174	3.3
Q61024	Asparagine synthetase (glutamine-hydrolyzing)	Asns	5	12.0815	0.0172	3.2
Q7TPZ8	Carboxypeptidase A1	Cpa1	2	9.3357	0.0239	3
Q9CR35	Chymotrypsinogen B	Ctrb1	2	4.0493	0.0361	3
D3Z6P0	Protein disulfide-isomerase A2	Pdia2	11	34.7523	0.0173	2.9
P21614	Vitamin D-binding protein	Gc	4	10.5449	0.0298	2.9
Q61838	Pregnancy zone protein	Pzp	11	30.1861	0.0268	2.8
P15947	Kallikrein-1	Klk1	3	7.0787	0.0276	2.8
P49290	Eosinophil peroxidase	Epx	5	13.9853	0.0169	2.7
P07758	Alpha-1-antitrypsin 1-1	Serpina1	4	13.4458	0.0233	2.5
P23953	Carboxylesterase 1C	Ces1c	3	10.9205	0.0292	2.5
Q8VDJ3	Vigilin	Hdlbp	2	5.4235	0.0301	2.5
P50172	Corticosteroid 11-beta-dehydrogenase isozyme 1	Hsd11b1	2	5.8113	0.0207	2.3
Q921I1	Serotransferrin	Tf	9	25.5997	0.0268	2.2
Q00897	Alpha-1-antitrypsin 1-4	Serpina1d	3	9.79536	0.0314	2
Q64511	DNA topoisomerase 2-beta	Top2b	2	4.0629	0.0477	1.9
P15626	Glutathione S-transferase Mu 2	Gstm2	3	10.3769	0.0340	1.8
Q9D855	Cytochrome b-c1 complex subunit 7	Uqcrb	3	8.1900	0.0126	1.8
Q8R2E9	ERO1-like protein beta	Ero1b	2	4.7424	0.0168	1.8
Q62087	Serum paraoxonase/lactonase 3	Pon3	4	13.6453	0.0167	1.7
Q99K85	Phosphoserine aminotransferase	Psat1	4	11.5566	0.0174	1.7
Q9DBF1	Alpha-aminoadipic semialdehyde dehydrogenase	Aldh7a1	4	10.4946	0.0308	1.7
O08807	Peroxiredoxin-4	Prdx4	3	8.6493	0.0172	1.7
P61620	Protein transport protein Sec61 subunit alpha isoform 1	Sec61a1	3	11.7206	0.0167	1.7
Q06138	Calcium-binding protein 39	Cab39	2	5.5638	0.0338	1.7
P10852	4F2 cell-surface antigen heavy chain	Slc3a2	4	12.8087	0.0122	1.6
P09103	Protein disulfide-isomerase	P4hb	4	10.0713	0.0189	1.6
P58252	Elongation factor 2	Eef2	3	9.4214	0.0256	1.6
Q6ZWQ7	Signal peptidase complex subunit 3	Spcs3	2	4.2641	0.0210	1.6
P35564	Calnexin	Canx	2	5.9499	0.0312	1.6
Q78PY7	Staphylococcal nuclease domain-containing protein 1	Snd1	5	13.1260	0.0295	1.5
O35855	Branched-chain-amino-acid aminotransferase, mitochondrial	Bcat2	2	6.0829	0.0174	1.5
Q64674	Spermidine synthase	Srm	2	5.6741	0.0172	1.5
Q922Q8	Leucine-rich repeat-containing protein 59	Lrrc59	2	4.9835	0.0170	1.5
Q922R8	Protein disulfide-isomerase A6	Pdia6	2	6.9604	0.0234	1.5

**Table 5:** Increased Proteins in the *mdx-4cv* Spleen as Determined by LC-MS/MS Analysis

Accession	Protein	Gene	Unique Peptides	Confidence Score	Adjusted p Value	Max Fold Change
P21981	Protein-glutamine gamma-glutamyl-transferase 2	Tgm2	2	4.74003	0.0124	3.8
P01878	Ig alpha chain C region	–	2	5.5789	0.0332	2.9
Q8CJ91	CD209 antigen-like protein B	Cd209b	2	5.1658	0.0152	2.5
P41245	Matrix metalloproteinase-9	Mmp9	2	5.9333	0.0175	2.3
P08071	Lactotransferrin	Ltf	15	49.8443	0.0136	2.1
P54869	Hydroxymethylglutaryl-CoA synthase, mitochondrial	Hmgcs2	3	7.7425	0.0130	2
P21300	Aldo-keto reductase family 1 member B7	Akr1b7	2	5.8779	0.0183	2
P50608	Fibromodulin	Fmod	2	4.9599	0.0190	1.9
Q3UMY5	Echinoderm microtubule-associated protein-like 4	Eml4	2	6.1649	0.0120	1.9
P82343	N-acylglucosamine 2-epimerase	Renbp	2	6.3056	0.0170	1.9
O08692	Neutrophilic granule protein	Ngp	2	5.4646	0.0185	1.9
P26039	Talin-1	Tln1	2	5.7934	0.0181	1.8
Q9CWK8	Sorting nexin-2	Snx2	2	6.9348	0.0183	1.8
Q8VEE1	LIM and cysteine-rich domains protein 1	Lmcd1	2	6.8011	0.0085	1.8
P19070	Complement receptor type 2	Cr2	2	5.0366	0.0295	1.8
P27870	Proto-oncogene vav	Vav1	3	7.4085	0.0192	1.7
Q9ES52	Phosphatidylinositol 3,4,5-trisphosphate 5-phosphatase 1	Inpp5d	3	8.2105	0.0152	1.7
Q8R1G6	PDZ and LIM domain protein 2	Pdlim2	2	6.4459	0.0286	1.7
Q6P8X1	Sorting nexin-6	Snx6	2	6.9493	0.0176	1.7
P49710	Hematopoietic lineage cell-specific protein	Hcls1	2	6.3795	0.0295	1.7
P63038	60 kDa heat shock protein, mitochondrial	Hspd1	2	4.9004	0.0312	1.7
Q61823	Programmed cell death protein 4	Pdcd4	2	5.2918	0.0147	1.7
Q6P4T2	U5 small nuclear ribonucleoprotein 200 kDa helicase	Snrnp200	2	6.1287	0.0154	1.7
Q62230	Sialoadhesin	Siglec1	6	19.4232	0.0124	1.6
Q8VCW8	Medium-chain acyl-CoA ligase ACSF2, mitochondrial	Acsf2	5	16.0841	0.0118	1.6
Q9QVP9	Protein-tyrosine kinase 2-beta	Ptk2b	3	11.6718	0.0265	1.6
Q8K2T1	NmrA-like family domain-containing protein 1	Nmral1	3	9.4830	0.0122	1.6
Q921T2	Torsin-1A-interacting protein 1	Tor1aip1	2	4.2835	0.0135	1.6
Q3UP87	Neutrophil elastase	Elane	2	4.6109	0.0260	1.6
Q99KQ4	Nicotinamide phosphoribosyltransferase	Nampt	2	6.6515	0.0164	1.6
Q9QXS1	Plectin	Plec	11	34.4355	0.0152	1.5
Q80SU7	Gvin1	Gvin1	4	12.9185	0.0293	1.5
P05555	Integrin alpha-M	Itgam	4	11.3340	0.0157	1.5
Q62261	Spectrin beta chain, non-erythrocytic 1	Sptbn1	3	10.1324	0.0265	1.5
O70318	Band 4.1-like protein 2	Epb41l2	2	4.2166	0.0138	1.5
P23475	X-ray repair cross-complementing protein 6	Xrcc6	2	6.1252	0.0180	1.5
Q9CQE5	Regulator of G-protein signaling 10	Rgs10	2	5.1948	0.0124	1.5
P55194	SH3 domain-binding protein 1	Sh3bp1	2	7.2682	0.0149	1.5

**Figure 5:**
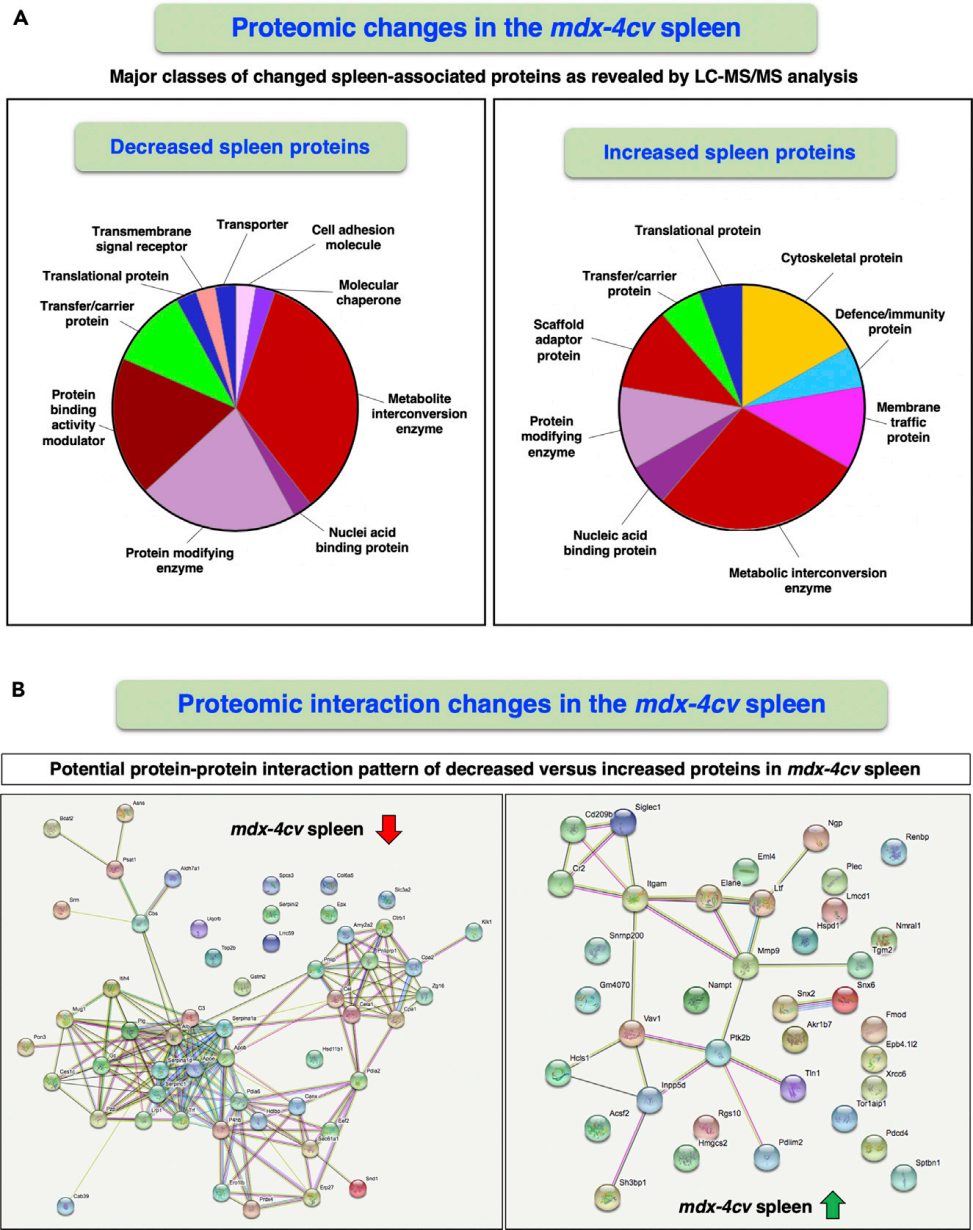
Bioinformatic Analysis of Proteomic Changes in the *mdx-4cv* Spleen Overview of the distribution of changed protein classes and potential protein-protein interaction patterns of decreased versus increased proteoforms in the spleen from the *mdx-4cv* mouse model of Duchenne muscular dystrophy, as determined by the bioinformatics software programs PANTHER (A) and STRING (B), respectively.

**Figure 6:**
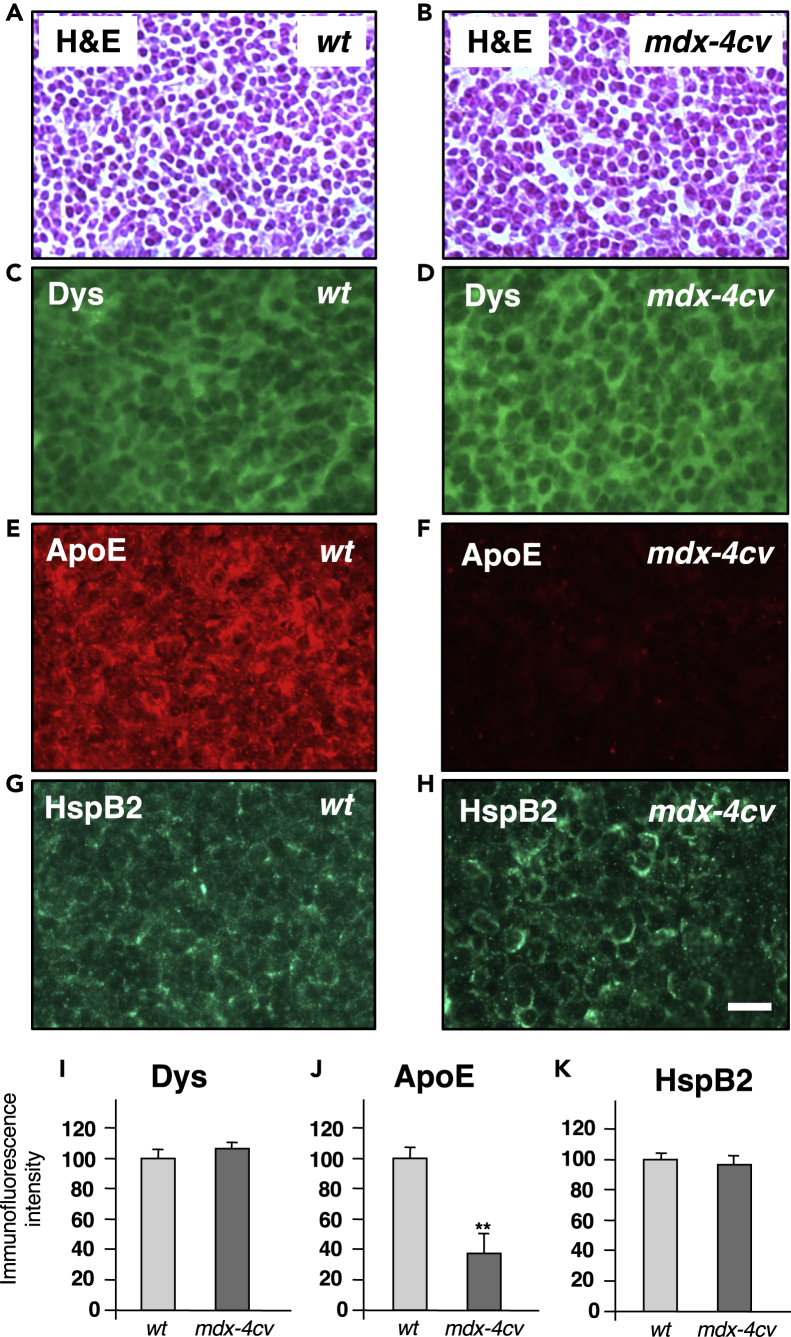
Histological and Immunofluorescence Microscopical Characterization of the Spleen from the *mdx-4cv* Mouse Model of Duchenne Muscular Dystrophy (A–H) Shown are transverse cryosections of wild-type (*wt*) (A, C, E, and G) and *mdx-4cv* (B, D, F, and H) spleen stained with hematoxylin and eosin (H&E) (A and B) and labeled with antibodies to dystrophin (Dys) (C and D), apolipoprotein ApoE (E and F), and the molecular chaperone HspB2 (G and H). (I–K) Analysis of immunofluorescence intensities (unpaired Student's t test; mean values ± SEM; n = 4; ∗∗p < 0.01). Scale bar, 25 μm.

The bioinformatic PANTHER and STRING analyses depicted in Figure 5 summarize the proteome-wide changes in the *mdx-4cv* spleen, as well as potentially altered protein-protein interaction patterns of changed spleen-associated proteins. Both considerable decreases and increases were observed for metabolic interconversion enzymes, such as hydrolases, ligases, lyases, oxidoreductases, and transferases. Striking increases included protein modifiers such as proteases, protein modulators such as protease inhibitors, and transfer carriers. In relation to altered protein interactions, especially striking is the apolipoprotein hub with ApoE and ApoB of reduced protein species. Immunofluorescence microscopy clearly confirmed the drastic reduction of ApoE in the *mdx-4cv* spleen (Figures 6E, 6F, and 6J) and indicated that the expression of the short spleen-associated isoform of dystrophin is not affected in dystrophinopathy (Figures 6C, 6D, and 6I). No major histological changes (Figures 6A and 6B) and comparable expression levels of the molecular chaperone HspB2 (Figures 6G, 6H, and 6K) were observed in the *mdx-4cv* spleen.

**Figure 7:**
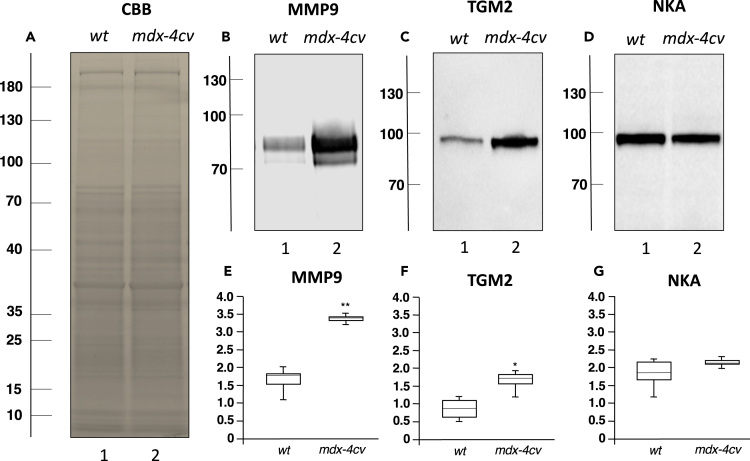
Immunoblot Analysis of the Spleen from the *mdx-4cv* Mouse Model of Duchenne Muscular Dystrophy (A–D) Shown is a protein gel of wild-type (*wt*) versus *mdx-4cv* spleen samples (A) and identical nitrocellulose replicas labeled with an antibody to matrix metalloproteinase MMP9 (B), transglutaminase TGM2 (C), and Na^+^/K^+^-ATPase (D). Lanes 1 and 2 contain wild-type and *mdx-4cv* specimens, respectively. (E–G) In panels (E–G) the box plots of the immunoblot analysis are shown (Mann-Whitney U test; n = 7; ∗p < 0.05; ∗∗p < 0.01). The values of molecular mass standards (x10^−3^ kDa) are marked on the left side of the gel and immunoblots.

Potentially excreted forms of proteins were identified as transglutaminase 2 (protein-glutamine gamma-glutamyl-transferase TGM2) and matrix metalloproteinase MMP9. The protein band pattern of gel electrophoretically separated spleen samples did not show major differences between wild-type and *mdx-4cv* preparations (Figure 7A). However, the increased abundance of TGM2 and MMP9 in the *mdx-4cv* spleen was confirmed by immunoblotting (Figures 7B, 7C, 7E, and 7F). In contrast, the spleen-associated alpha-subunit of the Na^+^/K^+^-ATPase showed no major changes in its density in the dystrophic phenotype (Figures 7D and 7G).

## Discussion

### The *mdx-4cv* Mouse Model of Dystrophinopathy

Spontaneous or bioengineered *mdx*-type mouse models of dystrophinopathy have been widely used in muscular dystrophy research (Partridge, 2013; Wilson et al., 2017), and there are ongoing efforts to improve specific aspects of dystrophic mice to better mirror the human pathology (Yucel et al., 2018). The *mdx-4cv* model has been successfully employed for the evaluation of experimental therapeutic approaches for the restoration of dystrophin (Mitrpant et al., 2009; Kimura et al., 2010; Bengtsson et al., 2017) and the systematic identification of novel biomarker candidates to improve differential diagnosis, prognosis, and therapy monitoring (Hathout et al., 2014; Dowling et al., 2019). The cell biological characterization of the *mdx-4cv* mouse, which was generated by chemical mutagenesis (Chapman et al., 1989) and is characterized by a 10-fold lower rate of Dp427-positive revertant muscle fibers as compared with the naturally occurring *mdx-23* mouse (Danko et al., 1992), has established distinct pathological alterations throughout the lifetime of this animal model (Latroche et al., 2015). This includes varying degrees of fiber degeneration, reactive myofibrosis, sterile inflammation, and metabolic disturbances in limb muscles (Murphy et al., 2019a), the diaphragm (Murphy et al., 2019b), and the heart (Murphy et al., 2016), as well as proteome-wide alterations in the brain (Murphy et al., 2015), the liver (Murphy et al., 2018a), and the kidney (Dowling et al., 2020b). The concentration of distinct protein biomarkers is also markedly affected in *mdx-4cv* serum (Murphy et al., 2017), saliva (Murphy et al., 2018b), and urine (Gargan et al., 2020). These complex and body-wide changes make the *mdx-4cv* mouse a suitable model system to study the molecular pathogenesis of dystrophinopathy.

### The Spleen Proteome and Muscular Dystrophy

The comparative mass spectrometric analysis presented in this report is based on an excellent coverage of the experimentally accessible spleen proteome in wild-type versus *mdx-4cv* extracts. Previous large-scale surveys using both systematic mass spectrometric and antibody-based methodology have established a wide range of spleen-associated proteins (Andersson et al., 2014; Dudekula and Le Bihan, 2016; Goltsev et al., 2018; Thul and Lindskog, 2018; Uhlén et al., 2015; Wilhelm et al., 2014). Many of these key marker proteins of the spleen were identified by a high degree of sequence coverage by unique peptides in this study. In agreement with established splenic changes in dystrophic animal models of Duchenne muscular dystrophy, such as morphological adaptations in the white pulp domain of the spleen (Santos et al., 2013) and altered levels of immune cells in the spleen and enhanced migration of inflammatory cells from the splenic reservoir to dystrophic muscle tissues (Farini et al., 2016; Giordano et al., 2015; Mojumdar et al., 2014, 2016; Ouisse et al., 2019), this investigation has established considerable effects on the spleen proteome owing to deficiency in the full-length dystrophin isoform Dp427-M. Since the spleen acts as a dominant reservoir for inflammatory cells (Ingersoll et al., 2011) and splenic monocytes were recently shown to play an important role during chronic inflammation of dystrophic fibers (Rizzo et al., 2020), the novel proteomic findings are in agreement with the pathophysiological idea of a connection between the lymphoid system and dystrophic muscles with an inflammatory phenotype (Villalta et al., 2015; Tidball et al., 2018).

### Preserved Expression of a Short Isoform of Dystrophin in the *mdx-4cv* Spleen

X-linked muscular dystrophy is a complex neuromuscular disorder that is characterized by necrosis, myofibrosis, and inflammation in the skeletal musculature, as well as late-onset cardiomyopathy, respiratory impairments, neurological deficiencies, scoliosis, and metabolic disturbances (Mercuri et al., 2019; Thompson et al., 2020). The neuromuscular symptoms are due to primary abnormalities in the extremely large *DMD* gene, which encodes several isoforms of the protein dystrophin (Muntoni et al., 2003). The proteomic survey of the spleen presented here has identified unique peptides that belong to the dystrophin protein sequence. This not-well-characterized spleen-associated proteoform of the dystrophin protein is most likely a short version of the *DMD* gene product, such as the ubiquitously expressed Dp71-G isoform (Tokarz et al., 1998). The proteomic analysis found the presence of this dystrophin isoform in all analyzed spleen samples with high confidence; thus, the nonsense mutation in exon 53 of the *DMD* gene does not appear to affect the production of this short dystrophin isoform in the *mdx-4cv* mouse (Im et al., 1996). However, immunofluorescence microscopy clearly showed that the full-length Dp427-M isoform of dystrophin is absent from dystrophic *mdx-4cv gastrocnemius* muscle fibers (Murphy et al., 2019a), confirming the dystrophic phenotype.

### Proteomic Identification of Dystrophin-Associated Proteins in the Spleen

The full-length isoform of dystrophin, Dp427-M, functions in contractile fibers as a membrane cytoskeletal component and forms a supramolecular assembly with a variety of sarcolemma-associated proteins. The dystrophin core complex, consisting of Dp427-M, dystroglycans, sarcoglycans, dystrobrevins, syntrophins, and sarcospan, links the extracellular matrix component laminin to the intracellular actin cytoskeleton (Ohlendieck, 1996). This trans-plasmalemmal structure plays a key role in lateral force transmission and the stabilization of the fiber surface during excitation-contraction-relaxation cycles (Murphy and Ohlendieck, 2015). In dystrophinopathy, the almost complete loss of Dp427-M causes a drastic reduction in the members of the dystrophin-associated glycoprotein complex, which in turn triggers sarcolemmal micro-rupturing and calcium-induced proteolytic degradation (Guiraud et al., 2015). Here, we have extended the characterization of dystrophin-associated glycoproteins to the spleen and have identified by mass spectrometry the presence of dystroglycan, sarcoglycans, dystrobrevin, and syntrophins in this crucial secondary lymphoid organ.

### Drastic Reduction of Apolipoproteins and Related Serum Proteins in the *mdx-4cv* Spleen

A variety of complex changes in the immunobiology of dystrophic skeletal muscles have been established that might be reflected by variations in the lymphoid system (Villalta et al., 2015; Lozanoska-Ochser et al., 2018; Tidball et al., 2018). The most striking finding of this investigation is the drastic reduction in a variety of serum proteins in the *mdx-4cv* spleen. This includes proteins that are involved in lipid transport and metabolism (apolipoproteins ApoB-100 and ApoE, lipoprotein receptor Lrp1, and various lipases), the complement system (complement C3), metabolite transportation (albumin), digestion (amylase), and factors involved in the acute response and inflammation (kallikrein, inter alpha-trypsin inhibitor). Spleen-associated proteins with a drastic reduction in the *mdx-4cv* mouse model were previously shown to be also reduced in *mdx-4cv* serum (Murphy et al., 2017). This included the most significantly decreased protein species in the *mdx-4cv* spleen, apolipoprotein B-100, as well as antithrombin-III, complement C3, alpha-1-antitrypsin, murinoglobulin-1, plasminogen, inter-alpha-trypsin inhibitor heavy chain, chymotrypsin-like elastase, vitamin D-binding protein, and serotransferrin.

Apolipoproteins are crucial plasma proteins involved in the regulation of lipid homeostasis, and their concentration is a good indicator of the metabolic status of the organism (Geyer et al., 2017). Surprisingly, in the ApoE-null *mdx* mouse reduced atherosclerotic plaque formation was found, as well as decreased numbers of CD3+ cells in the spleen (Shami et al., 2015). In the case of the *mdx-4cv* model of dystrophinopathy, the reduced abundance of these plasma proteins in the spleen could reflect their lower concentration in the circulatory system. Impaired blood flow due to weakened cardiac output of the dystrophin-deficient heart might also be a crucial factor that causes a decrease in plasma components in the *mdx-4cv* spleen. In dystrophinopathy, late-onset cardiomyopathy causes cardiac weakness (Finsterer and Cripe, 2014) and this is reflected by drastic proteome-wide changes especially at the level of metabolic enzymes (Murphy et al., 2016). This cardiac impairment could cause a decreased rate of circulation of blood and concomitant chronic lack of proper supply of oxygen and nutrients to peripheral organs. The proteomic findings on the *mdx-4cv* spleen presented here agrees with this pathophysiological concept.

### Increased Transglutaminase TGM2 and Matrix Metalloproteinase MMP9 in the *mdx-4cv* Spleen

The most increased protein in the *mdx-4cv* spleen was identified as protein-glutamine gamma-glutamyl-transferase TGM2, also more commonly referred to as transglutaminase 2, which is a multi-functional enzyme that mediates the cross-linking of proteins and catalyzes the conjugation of polyamines to proteins (Beninati et al., 2017). In addition, more recently transglutaminase 2 was shown to be intrinsically involved in the maintenance of proteostasis (D'Eletto et al., 2019). Since chronic cellular stress results in the increased occurrence of abnormal protein folding or detrimental protein aggregation, the regulation of chaperone function is upregulated under dystrophic conditions (Doran et al., 2006; Brinkmeier and Ohlendieck, 2014). Thus, the elevated levels of transglutaminase TGM2 might be involved in a key aspect of the regulation of protein homeostasis in the stressed *mdx-4cv* spleen. The increased abundance of the matrix metalloproteinase MMP9 agrees with the findings from the previous screening of serum from the *mdx* mouse and patients with Duchenne muscular dystrophy for minimally invasive biomarker candidates (Nadarajah et al., 2011; Hathout et al., 2014; Anaya-Segura et al., 2015). The serum-associated proteoform of this member of the large family of matrix metalloproteinases was shown to be increased significantly in muscular dystrophy, which might explain its elevated levels in the *mdx-4cv* spleen. Although MMP9 probably plays a differential role during the progression of muscle degeneration (Shiba et al., 2015), its primary effect in muscular dystrophy appears to be the promotion of inflammation, tissue remodeling, and reactive myofibrosis (Kherif et al., 1999; Hindi et al., 2013; Shin et al., 2013). This confirms MMP9 as a good biomarker candidate for secondary changes in muscular dystrophy due to sterile inflammation and fibrotic changes.

### Novel Biomarker Candidates of Muscular Dystrophy-Associated Changes in the Spleen

In conclusion, although the expression levels of a short spleen-associated dystrophin isoform and its associated proteins do not appear to be majorly affected in muscular dystrophy, the absence of the full-length dystrophin isoform in contractile tissues seems to trigger secondary effects in the lymphoid system. This includes a drastic reduction in the apolipoproteins ApoB-100 and ApoE and a concomitant increase in the enzyme protein-glutamine gamma-glutamyl-transferase TGM2 and the matrix metalloproteinase MMP9, which might be useful candidates as novel biomarkers of dystrophinopathy-related changes in the spleen (Dowling et al., 2019; Al-Khalili Szigyarto, 2020). The proteomic findings suggest that pathophysiological cross talk and/or anatomical interconnectivity affects the spleen in muscular dystrophy, which substantiates the appropriateness of the *mdx-4cv* mouse model for studying secondary changes in non-muscle tissues in dystrophinopathy.

### Limitations of the Study

In this report, we have characterized for the first time the secondary impact of dystrophin deficiency on the spleen and established considerable proteome-wide changes in this lymphoid organ. However, the study was carried out with a genetic animal model of dystrophinopathy, the *mdx-4cv* mouse, which shows a milder pattern of neuromuscular degeneration in most skeletal muscles as compared with patients with Duchenne muscular dystrophy. It will therefore be crucial to extend these studies in the future to the evaluation of secondary abnormalities in X-linked muscular dystrophy to patient specimens. Although comparative proteomics is an excellent bioanalytical approach to determine systematic changes in the dystrophic phenotype, mass spectrometric lists of altered protein expression levels do neither give detailed information on the pathobiochemical time course of cellular alterations nor provide a deep mechanistic understanding of disease progression. Thus, based on the comparative proteomic data supplied in this report, it will be crucial to further study the underlying disturbances that cause proteome-wide changes in the spleen in association with dystrophinopathy.

### Resource Availability

#### Lead Contact

For further information, requests should be directed to the Lead Contact: Kay Ohlendieck (kay.ohlendieck@mu.ie).

#### Materials Availability

This study did not generate new unique reagents.

#### Data and Code Availability

The proteomic datasets from the mass spectrometric analysis of 14 separate spleen sample runs have been deposited to the Open Science Framework repository as OSF entry “f85ve” under the following link: https://osf.io/f85ve/. This link also features a multi-consensus file of the proteomic cataloging of mouse spleen with the help of an Orbitrap Fusion Tribrid mass spectrometer.

## Methods

All methods can be found in the accompanying Transparent Methods supplemental file.

## Supplemental Information

Document S1. Transparent Methods
