# Supplementary material for: Proteome-wide Changes in the mdx-4cv Spleen due to Pathophysiological Cross Talk with Dystrophin-Deficient Skeletal Muscle
Source: iScience. 2020 Aug 26;23(9):101500. doi: 10.1016/j.isci.2020.101500 (PMC7490529; doi:10.1016/j.isci.2020.101500)
Supplement: Document S1. Transparent Methods [file mmc1.pdf]

iScience, Volume *23*

Supplemental Information

Proteome-wide Changes in the *mdx-4cv* Spleen due to Pathophysiological Cross Talk with Dystrophin-Deficient Skeletal Muscle

Paul Dowling, Stephen Gargan, Margit Zweyer, Michael Henry, Paula Meleady, Dieter Swandulla, and Kay Ohlendieck

Transparent Methods

Proteome-wide changes in the *mdx-4cv* spleen due to pathophysiological crosstalk with dystrophin-deficient skeletal muscle

Paul Dowling, Stephen Gargan, Margit Zweyer, Michael Henry, Paula Meleady, Dieter Swandulla and Kay Ohlendieck

MATERIALS AND METHODS

Materials

For the mass spectrometry-based proteomic analysis of spleen preparations, general materials and analytical grade reagents were obtained from Bio-Rad Laboratories (Hemel-Hempstead, Hertfordshire, UK), GE Healthcare (Little Chalfont, Buckinghamshire, UK) and Sigma Chemical Company (Dorset, UK). Protease inhibitors were purchased from Roche Diagnostics (Mannheim, Germany). Protein digestion was carried out with sequencing grade-modified trypsin, Lys-C and Protease Max Surfactant Trypsin Enhancer from Promega (Madison, WI, USA). The Pierce 660-nm Protein Assay reagents and Invitrogen NuPAGE Novex Bis-Tris gels were purchased from ThermoFisher Scientific (Dublin, Ireland). Vivacon 500 filter units were purchased from Sartorius (Göttingen, Germany). Expedeon InstantBlue Coomassie Protein Stain was from Abcam (Cambridge, MA, USA). Primary antibodies were purchased from Antibody Genie, Dublin, Ireland (CAB16344 against apolipoprotein ApoE), Cell Signaling Technology, Leiden, The Netherlands (mAb23565 against the alpha-subunit of the Na^+/K^+-ATPase), Abbexa Ltd, Cambridge, UK (abx001907 against heat shock protein HspB2; abx000934 against transglutaminase TGM2), Leica Biosystems, Wetzlar, Germany (NCL-

DYS1 and NCL-DYS2 against dystrophin) and R&D Systems, Abingdon, UK (AF909 against matrix metalloproteinase MMP9; MAB4376 against transglutaminase TGM2). Anti-rabbit and anti-mouse peroxidase conjugated secondary antibodies were from Cell Signaling Technology (Leiden, The Netherlands). Normal goat serum, goat anti-mouse IgG RRX (Rhodamine Red-X), anti-rabbit Alexa Fluor 488 and Alexa 647 antibodies were purchased from Molecular Probes, Life Technologies (Darmstadt, Germany) and Jackson ImmunoResearch (West Grove, PA, USA), respectively. The embedding medium Fluoromount G was from Southern Biotech (Birmingham, AL, USA).

Preparation of mouse tissue extracts for proteomic and biochemical analysis

Mice were obtained from the Bioresource Unit of the University of Bonn (Murphy et al., 2019a), where they were kept under standard conditions and all procedures adhered to German legislation on the use of animals in experimental research (Amt für Umwelt, Verbraucherschutz und Lokale Agenda der Stadt Bonn, North Rhine-Westphalia, Germany). The spleen and *gastrocnemius* muscle from 12-month old male wild type C57/BL6 mice and age-matched dystrophic male *mdx-4cv* mice were freshly dissected and quick-frozen in liquid nitrogen. Tissue specimens were transported to Maynooth University in accordance with the Department of Agriculture (animal by-product register number 2016/16 to the Department of Biology, National University of Ireland, Maynooth) on dry ice and stored at -80ºC prior to proteomic and biochemical analysis. For sample preparation, 25 mg of mouse tissues (wild type versus dystrophic phenotype) were lysed by homogenisation with 200µl of lysis solution (4% SDS, 100mM Tris-Cl pH 7.6, 0.1M dithiothreitol) and incubated at 95°C for 3 minutes and then sonicated for 30 seconds. The lysate was clarified by centrifugation at 16,000x*g* for 5 minutes. Tissue aliquots were used for gel electrophoretic separation and comparative immunoblotting. Spleen extracts were further processed for mass spectrometry. 10µl of lysate was mixed with

200µl of 8M urea, 0.1M Tris pH 8.9 in filter units (Sartorius, Vivacon 500, Product number: VN0H22) and centrifuged at 14,000x*g* for 15 minutes. Samples were subsequently processed according to the standardized FASP protocol for filter-aided sample preparation (Wiśniewski, 2009).

Label-free liquid chromatography mass spectrometry

For the proteomic profiling of the *mdx-4cv* spleen, reverse-phased capillary high-pressure liquid chromatography was performed with the help of the UltiMate 3000 nano system from Thermo Scientific coupled directly in-line with the Thermo Orbitrap Fusion Tribrid Mass Spectrometer (Murphy et al., 2019b). The digested protein samples (1µl ~ 800ng peptide) were loaded onto the trapping column (PepMap100, C18, 300 µm × 5 mm) at a flow rate of 25 µl/min with 2% (v/v) acetonitrile (ACN), 0.1% (v/v) trifluoroacetic acid (TFA) for 3 minutes before being resolved onto an analytical column (Acclaim PepMap 100, 75 µm × 50 cm, 3 µm bead diameter column). Peptides were eluted using the following binary gradient; solvent A (0.1% (v/v) formic acid in LC-MS grade water) and solvent B (80% (v/v) ACN, 0.08% (v/v) formic acid in LC-MS grade water) using 2-23% B for 75 minutes, 32-90% B for 5 minutes and holding at 90% B for 5 minutes at a flow rate of 300 nl/min. For peptide ionization, a voltage of 2.0 kV was applied and a capillary temperature of 320°C was used. Data-dependent acquisition with full scans in the 380-1500 m/z range was performed using an Orbitrap mass analyser with a resolution of 120,000 (at m/z 200), a targeted automatic gain control (AGC) value of 4E+05 and a maximum injection time of 50ms. The number of selected precursor ions for fragmentation was determined by the top-speed acquisition algorithm. Selected precursor ions were isolated in the Quadrupole with an isolation width of 1.6 Da. Peptides with a charge state of 2+ to 7+ were analysed and a dynamic exclusion was applied after 60s. Precursor ions were fragmented using higher energy collision-induced dissociation with a normalized

collision energy of 28%, and resulting MS/MS ions were measured in the linear ion trap. The typical MS/MS scan conditions were as follows: a targeted AGC value of 2E+04 and a maximum fill time of 35ms.

Data analysis

Qualitative data analysis, using mass spectrometric files (.raw), was performed by searching against the UniProtKB-SwissProt *Mus musculus* database with Proteome Discoverer 2.2 using Sequest HT (Thermo Fisher Scientific), and Percolator. The following search parameters were used for protein identification: (i) peptide mass tolerance set to 10 ppm, (ii) MS/MS mass tolerance set to 0.02 Da, (iii) an allowance of up to two missed cleavages, (iv) carbamido-methylation set as a fixed modification and (v) methionine oxidation set as a variable modification. Peptides were filtered using a minimum XCorr score of 1.5 for 1, 2.0 for 2, 2.25 for 3 and 2.5 for 4 charge states, with peptide probability set to high confidence (Qu et al, 2017).

Quantitative label-free data analysis was performed using Progenesis QI for Proteomics (version 2.0; Nonlinear Dynamics, a Waters company, Newcastle upon Tyne, UK), essentially as recommended by the manufacturer (www.nonlinear.com). Peptide and protein identification from this analysis were achieved with Proteome Discoverer 2.2 using Sequest HT (Thermo Fisher Scientific), and Percolator as described above and were imported into Progenesis QI software for further analysis.

Protein identifications were reviewed, and only those which passed the following criteria were considered differentially expressed between experimental groups with high confidence and statistical significance: (i) an ANOVA p-value of ≤0.01 between experimental groups; (ii) proteins with ≥2 unique peptides contributing to the identification. To calculate the maximum fold change for a protein, Progenesis QI calculates the mean abundance for that protein in each

experimental condition. These mean values are then placed in a condition-vs-condition matrix to find the maximum fold change between any two condition's mean protein abundances. The freely available software packages PANTHER (Mi et al., 2017; http://pantherdb.org/) and STRING (Szklarczyk et al., 2019; https://string-db.org/) were used to identify protein classes and characterise potential protein interactions, respectively.

Gel electrophoresis and immunoblotting

Protein fractions (12.5µg protein per lane for Coomassie Blue staining; and 25µg protein per lane for immunoblot analysis) were run on NuPAGE Novex Bis-Tris Gels under standard conditions (Murphy et al., 2019a). Protein concentration was determined using the Pierce 660-nm protein assay system (Antharavally et al., 2009). For comparative analyses, Coomassie staining of protein gels was carried out with InstantBlue Coomassie Protein Stain. Immunoblotting was then employed as an orthogonal method for the independent verification of changes in newly identified proteins in the *mdx-4cv* spleen. Proteins were transferred to nitrocellulose membranes, blocked and incubated with primary antibody overnight, followed by detection with peroxidase-conjugated secondary antibodies using the enhanced chemiluminescence method (Murphy et al., 2019a). Densitometric scanning and statistical analysis of immunoblots was performed using a HP PSC-2355 scanner and ImageJ software (NIH, Bethesda, MD, USA) along with Microsoft Excel in which statistical significance was based on a p-value ≤ 0.05.

Histological analysis and immunofluorescence microscopy

Tissue cryo-sections from skeletal muscle and spleen were used for standardized histological and immunofluorescence microscopical analysis (Dowling et al., 2020). The mutant status of the *mdx-4cv* mouse was established by demonstrating the loss of the dystrophin isoform

Dp427-M in *gastrocnemius* muscle and correlated to potential changes in the spleen. Histological features of the analysed tissues were visualized by haematoxylin and eosin staining. For immunofluorescence microscopy, freshly dissected spleen and skeletal muscle specimens from 12-month old wild type and *mdx-4cv* mice were quick-frozen in liquid nitrogen-cooled isopentane and 10µm sections cut in a cryostat. For dystrophin immuno-staining, unfixed cryosections were boiled in phosphate-buffered saline for 5 minutes as previously described in detail (Murphy et al., 2019b). For immuno-staining, sections were fixed in a 1:1 (v/v) mixture of methanol and acetone for 10 minutes at room temperature and then blocked with 1:20 diluted normal goat serum for 30 minutes at room temperature. Primary antibodies were diluted appropriately in phosphate-buffered saline for overnight incubation at 4°C. Tissue specimens were carefully washed and then incubated with fluorescently labelled secondary antibodies, using either 1:200 diluted anti-rabbit Alexa Fluor 488 or 647 antibodies or 1:200 diluted anti-mouse RRX antibody for 45 minutes at room temperature. Nuclei were counter-stained with 1µg/ml bis-benzimide Hoechst 33342. Antibody-labelled spleen and skeletal muscle sections were embedded in Fluoromount G medium and viewed under a Zeiss Axioskop 2 epifluorescence microscope equipped with a digital Zeiss AxioCam HRc camera (Carl Zeiss Jena GmbH, Jena, Germany). Immunofluorescence intensity was analysed using ImageJ software (NIH, Bethesda, MD, USA) along with Microsoft Excel in which statistical significance was based on a p-value ≤ 0.05.

References

Antharavally, B.S., Mallia, K.A., Rangaraj, P., Haney, P., and Bell, P.A. (2009) Quantitation of proteins using a dye-metal-based colorimetric protein assay, Anal. Biochem. *385*, 342-345.

Dowling, P., Zweyer, M., Raucamp, M., Henry, M., Meleady, P., Swandulla, D., and Ohlendieck, K. (2020). Proteomic and cell biological profiling of the renal phenotype of the *mdx-4cv* mouse model of Duchenne muscular dystrophy. Eur. J. Cell Biol. *99*, 151059.

Mi, H., Huang, X., Muruganujan, A., Tang, H., Mills, C., Kang, D., and Thomas, P.D. (2017). PANTHER version 11: expanded annotation data from Gene Ontology and Reactome pathways, and data analysis tool enhancements. Nucleic Acids Res. *45(D1)*, D183-D189.

Murphy, S., Zweyer, M., Henry, M., Meleady, P., Mundegar, R.R., Swandulla, D., and Ohlendieck, K. (2019a). Proteomic analysis of the sarcolemma-enriched fraction from dystrophic *mdx-4cv* skeletal muscle. J. Proteomics *191*, 212-227.

Murphy, S., Zweyer, M., Raucamp, M., Henry, M., Meleady, P., Swandulla, D., and Ohlendieck, K. (2019b). Proteomic profiling of the mouse diaphragm and refined mass spectrometric analysis of the dystrophic phenotype. J. Muscle Res. Cell. Motil. *40*, 9-28.

Qu, M., An, B., Shen, S., Zhang, M., Shen, X., Duan, X., Balthasar, J.P., and Qu, J. (2017). Qualitative and quantitative characterization of protein biotherapeutics with liquid chromatography mass spectrometry. Mass Spectrom. Rev. *36*, 734-754.

Szklarczyk, D., Gable, A.L., Lyon, D., Junge, A., Wyder, S., Huerta-Cepas, J., Simonovic, M., Doncheva, N.T., Morris, J.H., Bork, P., Jensen, L.J., and Mering, C.V. (2019). STRING v11: protein-protein association networks with increased coverage, supporting functional discovery in genome-wide experimental datasets. Nucleic Acids Res. *47(D1)*, D607-D613.

Wiśniewski, J.R. (2019). Filter Aided Sample Preparation - A tutorial. Anal. Chim. Acta *1090*, 23-30.
